# Supplementary material for: Two sugar beet chitinase genes, BvSP2 and BvSE2, analysed with SNP Amplifluor-like markers, are highly expressed after Fusarium root rot inoculations and field susceptibility trial
Source: PeerJ. 2018 Jun 27;6:e5127. doi: 10.7717/peerj.5127 (PMC6026450; doi:10.7717/peerj.5127)
Supplement: Supplemental Information 2 [file peerj-06-5127-s002.pdf]

Supplementary Table S1

List and origins of the 22 breeding lines and the reference DH line KWS2320

N	Breeding lines ID	Origins
1	1002	Breeding line from hybrid with Ramonskaya, Russia
2	1005	Breeding line from hybrid with Yaltushkovskaya, Ukraine
3	1017	Breeding line selected from Veselopodolyanskaya, Ukraine
4	1042	Breeding line from hybrid with Ramonskaya, Russia
5	1082	Breeding line from hybrid with Biyskaya, Russia
6	2115	Breeding line from hybrid with Nemerchanskaya, Ukraine
7	2125	Breeding line from hybrid with Ramonskaya, Russia
8	2154	Breeding line from hybrid with Pervomaiskaya, Russia
9	2172	Breeding line from hybrid with Ramonskaya, Russia
10	2182	Breeding line from hybrid with Biyskaya, Russia
11	2190	Breeding line from hybrid with Ramonskaya, Russia
12	2210	Breeding line from hybrid with Veselopodolyanskaya, Ukraine
13	2217	Breeding line selected from Veselopodolyanskaya, Ukraine
14	2236	Breeding line from hybrid with Kazakhstanskiy, Kazakhstan
15	2261	Breeding line from hybrid with Kazakhstanskiy, Kazakhstan
16	2262	Breeding line from hybrid with Ramonskaya, Russia
17	2263	Breeding line from hybrid with Nemerchanskaya, Ukraine
18	2281	Breeding line from hybrid with Kubanskiy, Russia
19	2282	Breeding line from hybrid with Belotzerkovskaya, Ukraine
20	2286	Breeding line from hybrid with Ligovskaya, Russia
21	2296	Breeding line from hybrid with Belotzerkovskaya, Ukraine
22	2300	Breeding line selected from Veselopodolyanskaya, Ukraine
23	KWS2320	Reference DH line KWS2320, KWS SAAT SE, Germany

Supplementary Figure S1

Sequence of the *BvSE2* gene, encoding chitinase, Class III, annotated for the reference DH line KWS2320

Start-codon (in green) and Stop-codon (in red) indicate an ORF of 882 bp. Primers for Sanger sequencing are shown in yellow with a fragment size of 1,076 bp. Position of SNP [W = A/T] with original allele 'A' from KWS2320 and SNP allele 'T' is indicated in dark blue. The gene-specific marker, KIZ4, contains two Forward primers with 3'-end on the SNP (shown in light blue) and common Reverse primer (shown in purple). The amplified product for SNP Amplifluor-like analysis is 81 bp.

>gi|731321417|ref|XM_010673039.1| PREDICTED: Beta vulgaris subsp. vulgaris acidic endochitinase SE2 (LOC104888158), mRNA KWS2320, chromosome 3

```
GAAACACTGTTTAAGTTTAGGATATAAATAGCATACATAGATCACGCGTTTTTTTAACAGGTACAAGAAT
TTTCTGTCTACCCTTCGGGTACTAAGACTACGCCCAGCATGTCCTTCCGTGAAGAGGAAGAGCAATGCTG
CCAACTGAGCTAGAGGCCTACAACTCATTGGTAGGAGGATCCTTATTTTTGTTCAGCATACTGTAGAGCC
CAATTCCAGAAAGTATCCCTACAGCATTTAAGCTTCTGCATTCCCATTTGTAGAAAAAGAGCCTCTTTGA
CTAGACTTTCCAAGCAAACTCTTTTAGACAATTTCCTCCCACTTTACAGCCTTTCGAATACGCAAAACTC
ATTGACTCATTGACTCTAACATCTATAAATTTCCTACTTACCCAACCTTAAATCTCACAACCCAAGCACT
GCATTGCATAGTTGCACATACGTACCCAAAACAAGCATGGCAGCCAAAATAGTGTCAGTTCTATTCCTGA
TTTCTCTCTTAATCTTTGCTTCATTCGAGTCCTCTCATGGCTCCCAAATTGTCATATACTGGGGCCAAAA
TGGTGATGAAGGAAGTCTTGCTGACACTTGTAACTCCGGAAACTACGGTACCGTGATCCTAGCTTTCGTA
GCTACCTTTGGTAACGGGCAAACCCCGGCGCTGAACTTAGCTGGGCACTGTGACCCTGCTACAAATTGTA
ACAGTCTGAGCAGTGACATCAAAACATGCCAACAGGCAGGCATTAAGGTACTCCTCTCTATAGGAGGTGG
TGCCGGAGGCTATTCTCTTTCCTCAACCGATGATGCAAACACATTTGCTGATTACCTCTGGAWCACTTAT
CTTGGGGGTCAGTCCAGCACCCGACCCCTTGGAGATGCAGTTTTGGATGGTATTGATTTCGATATCGAGA
GTGGTGATGGCAGATTTTGGGATGACCTAGCTAGAGCATTGGCAGGTCATAACAATGGTCAGAAAACAGT
GTACTTATCAGCAGCTCCTCAATGTCCCTTCCCAGATGCCAGCTTAAGCACTGCCATAGCCACAGGCCTA
TTCGACTATGTATGGGTTCAGTTCTACAACAACCCCCCTTGTCAATATGATACCAGCGCTGATAATCTCT
TGAGCTCGTGGAACCAGTGGACTACAGTACAAGCTAACCAGATCTTCCTCGGACTACCAGCATCAACTGA
TGCTGCCGGCAGTGGTTTTATTCCAGCAGATGCTCTTACATCTCAAGTCCTTCCCACTATCAAGGGTTCT
GCTAAATATGGAGGAGTCATGCTATGGTCAAAGGCATATGACAGTGGGTACAGCAGTGCTATTAAAAGCA
GTGTTTAATTTAAATTACTAGTGTATCCAAAGATATAGGTACAAAATAAGTTATAGAGATACATCAAAAA
ACCATCTTAGTTTTAAATTTTTTATGCACCACAAAAGCTTGTAATACTAATATACTATTATCATAAATGG
CTTATTGCCTCGCTATATTTTGGTGATTATTATATACACAGTTACAACTTCGCAA
```

Supplementary Figure S2

Sequence of the *BvSP2* gene, encoding chitinase, Class IV, annotated for the reference DH line KWS2320

Start-codon (in green) and Stop-codon (in red) indicate an ORF of 867 bp. Primers for Sanger sequencing are shown in yellow with fragment size of 1,609 bp. Position of SNP [Y = C/T] with original allele ‘C’ from KWS2320 and SNP allele ‘T’ is indicated in dark blue. The gene-specific marker, KIZ3, contains two Forward primers with 3’-end on the SNP (shown in light blue) and common Reverse primer (shown in purple). The size of amplified product for SNP Amplifluor-like analysis is 79 bp.

>gi|731323202|ref|XM_010673997.1| PREDICTED: Beta vulgaris subsp. vulgaris acidic endochitinase SP2 (LOC104888888), mRNA KWS2320, chromosome 3

```
AAATCACATAATCTTCTCATCAAAACAAAACTACCCAAAAAAAAATGGCACTTCTTTTGAAAAACACCTT
ATATTTAGCCTTAATCATCTCAGTAATCTCATCATTCCCAACTTCATTGTTTGCACAAAACTGTGGGTGT
GCCCCTAACCTATGCTGCAGCAAATTTGGGTTCTGTGGCACTGGAACACCTTATTGTGGTGTTGGTAACT
GTCAATCTGGCCCATGTGAGGGTGGCACTCCAACTACGCCGACAACTCCAACCACTCCTACTACGCCAGG
AACTGGTGGTGGTGGCTCATCAGTGTCTGATATAGTGAGTCAAGCATTCTTTGATGGGATTATAGGCCAA
GCTGCTGCATCTTGTCCTGGGAAAAACTTCTACACTCGYGCTGCTTTTCTTAGTGCTGTTGATCCTAAGT
TCGGAAACGAAGGTTCCAGTGATGATAACAAGAGAGAAATCGCTGCCTTCTTTGCTCATATCTCGCATGA
GACTACCAATTTGTGCCACATAGAAGAAAGAGATGGAGATGTTGGTGACGCCTACTGCGATCAAGACAAG
GCAGCTCAATACCCTTGCGCTGCAGGCAAGAAGTACTTCGGGAGAGGACCTCTTCAACTGTCATGGAACT
ACAACTATGCTCTTGCAGGACAGGCGATTGGATTTGATGGTCTTGGAAACCCTGAAAAGGTGGCTACTGA
TGTTAATACATCTTTCAAGGCTGCAATGTGGTTTTGGATGACTAATGTTCACTCCGTTATGAACCAAGGT
TTTGGAGCTACCACCAAAGCTATTAATGGCGCGCTTGAATGCAATGGCCAAAACCAGGATCAAGCCAATG
ACCGTATTCAGTTTTATAAGAAGTATTGTGCTGATTTTGGTGTTGCTCCTGGTGATAATCTTACATGTTA
ATTTCGACAAGTTACAAGTATATCTCAATATAGTACTTCTATATGATATATACATTCATGTTTATTAGTT
GCACTGAGGTATAATTTACTGAATAAAACACAGATACTATGTAGTACTAGAATGTTGTTCTTTCTTGATC
TTGCTATGAACAATGAATATATAATAAAAGGAATTACATGAGCATTTCTA
```

Supplementary Table S2

Primers used for qRT-PCR and their amplification products

Gene name	Primer name	Sequence (5’ – 3’)	Size of amplicon (bp)
Chitinase *SE2*	SE2-F	CCGGAGGCTATTCTCTTTCC	81
	SE2-R	ACTGACCCCCAAGATAAGTG	
Chitinase *SP2*	SP2-F	TTGATGGGATTATAGGCCAAG	79
	SP2-R	ACAGCACTAAGAAAAGCAGC	
Glutamine synthetase	GS-F	GACCTCCATATTACTGAAAGGAAG	118
	GS-R	GAGTAATTGCTCCATCCTGTTCA	
